# The impact of exclusive breastfeeding for 6 months and of complementary breastfeeding after 6 months on bone mineral density and bone remodeling

**DOI:** 10.1371/journal.pone.0328254

**Published:** 2025-08-04

**Authors:** Larissa Brazolotto Ferreira, Keny Gonçalves Tirapeli, Carla Cristiane Silva, José Eduardo Corrente, Tamara Beres Lederer Goldberg

**Affiliations:** 1 PhD of the Postgraduate Program in Tocogynecology, Botucatu Faculty of Medicine, Universidade Estadual Paulista (UNESP), Botucatu, São Paulo, Brazil; 2 Adjunct Professor of the Department of Human Movement Studies, Universidade Estadual de Londrina (UEL), Londrina, São Paulo, Brazil; 3 Adjunct Professor of the Department of Statistics of the Institute of Biosciences, UNESP - Botucatu, São Paulo, Brasil; 4 Full Professor of the Postgraduate Program in Tocogynecology, Senior Professor - UNESP, Faculty of Medicine of Botucatu, Universidade Estadual Paulista (UNESP), Botucatu, São Paulo, Brazil; American University of Beirut Medical Center, LEBANON

## Abstract

**Objective:**

The aim of this study was to evaluate the evolution of bone mass in exclusive breastfeeding mothers (EBF) for 6 months, and another cohort, who had also exclusively breastfed their children while concomitantly donating breast milk for 6 months (EBF+), with evaluation of their bone mass during the subsequent 6-month period of complementary breastfeeding.

**Methods:**

A group of exclusive breastfeeding mothers (n = 38) were evaluated at 15 days and six months postpartum, and a second group of EBF-donors (EBF+) (n = 39) were evaluated at six months and one year postpartum. Bone mineral content (BMC) and density (BMD) were evaluated by bone densitometry (DXA) and bone turnover markers were determined: osteocalcin (OC), bone alkaline phosphatase (BAP), and carboxy-terminal telopeptide (S-CTX).

**Results:**

There was a significant decrease in BMD in the lumbar spine (1.107 ± 0.109 and 1.075 ± 0.112 g/cm^2^; *p* < 0.001), total body (1.135 ± 0.086 and 1.119 ± 0.085 g/cm^2^; *p* < 0.001), and total proximal femur over the six months of EBF compared to the values obtained from the same EBF group at 15 days postpartum (mean percentage decreases in BMD of −3.4 ± 3.7% (*p* < 0.001) in lumbar spine, −2.5 ± 3.4% (*p* < 0.001) in total proximal femur, and −1.7 ± 1.9% (*p* = 0.001) in total body). For the group of EBF + who practiced complementary breastfeeding after 6 months, densitometric results indicated a tendency to incorporation of bone mass, with a mean percentage increase in BMD of 5.0 ± 3.9% for lumbar spine, and 1.6 ± 3.4% for total proximal femur.

**Conclusion:**

There was a significant physiological mobilization of bone mineral density in the lumbar spine, total body, and total proximal femur after six months of EBF. In the EBF+ group, even with continued complementary breastfeeding, densitometric results were higher than those observed at 6 months, indicating a continuous increase over time.

## Introduction

Breastmilk guarantees the necessary amounts of water, carbohydrates, lipids, and proteins for the adequate development of infants. Breastfeeding promotes the integral health of the mother/child binomial, with a great impact on Public Health [1,2]. The World Health Organization (WHO) and the United Nations Children’s Fund (UNICEF) recommend exclusive breastfeeding (EBF) without adding any type of food or liquids in the first six months of life [1,3].

In pregnancy and lactation, bone resorption increases to meet the calcium demands of the fetus and newborn [4]. Physiological adaptations related to bone mass during pregnancy result in increased intestinal absorption of calcium and an increase in the rate of maternal bone renewal. During lactation, there is a tendency to greater conservation of calcium at the renal level, however, the most relevant mechanism appears to be temporary maternal bone resorption [5].

During lactation, prolactin suppresses the hypothalamic-pituitary-ovarian axis, decreasing estradiol and progesterone levels, positively regulating the receptor activator of nuclear factor-kappa-B ligand (RANKL) and negatively regulating the release of osteoprotegerin by osteoblasts, which increases osteoclastic activity upstream, resulting in bone resorption [6–8].

Bone mineral density (BMD) obtained by Dual-energy X-ray absorptiometry (DXA) is considered the recommended assessment for the diagnosis of low bone mass and osteoporosis, however, this is a static measurement, that, when associated with the assessment of bone turnover markers, can result in an extremely dynamic assessment. The combination and complementation of both methods provide great potential in evaluations, bringing greater solidity to the diagnosis of loss or gain in bone mass [9].

The current study is justified by the lack of consistent information in the literature regarding the evolution of bone mass during the period of lactation. In a systematic review with meta-analysis [10], the results pointed to concerns, such as different experimental designs that made it difficult to compare results, indicating problems regarding the outcome of bone densities in EBF after six months of exclusive breastfeeding, with inadequate attention given to the inclusion of eutrophic, primiparous lactating women, control over vitamin D and calcium supplementation, and analysis using DXA in specific sites, so that the authors could confirm the real impact on bone mass after the exclusive lactation period. In the meta-analysis, only four studies, considered to have low risk of bias and good quality, were included [10].

Thus, the objective of the current study was to evaluate the bone mass in breastfeeding mothers by measuring bone mineral density and analyzing biochemical markers of bone formation and resorption, during the period of exclusive breastfeeding (EBF) and, for the continuity of complementary breastfeeding for six months, using results from a cohort of mothers who exclusively breastfed and also donated human milk (EBF+), with both groups being monitored prospectively (EBF and EBF+).

### Subjects and methods

**Fig 1:**
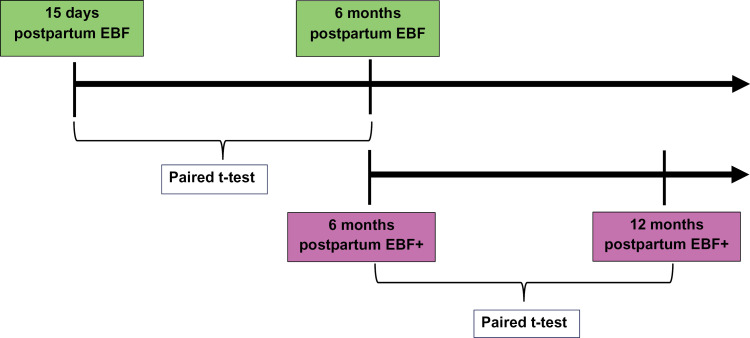
Timeline of monitoring of exclusive breastfeeding mothers (EBF) and exclusive breastfeeding – donors (EBF+).

This is a prospective, descriptive, and analytical longitudinal parallel study. One of the samples consisted of 38 women on their 15th day postpartum, recruited during postpartum consultations at Basic Health Units in the city of Araçatuba- SP, between January 6^th^, 2020 and December 30^th^, 2022. The exclusive breastfeeding mothers (EBF) evaluated at 15 days postpartum were the same mothers as evaluated at six months. In addition, during the same period, 39 human milk donors from the Human Milk Bank (HMB), in the same city, who also exclusively breastfed their children for six months and, in addition, donated excess breast milk during this period were monitored. This group donated an average of 9.4 ± 8.2 liters over 6 months (mean 52 ± 45mL/d), and stopped donating at 6 months postpartum, even though they continued breastfeeding, offering on average 6.1 ± 3.3 of breastfeeds per day, as well as introducing complementary foods. Densitometric variables and bone turnover markers were obtained in the sixth month of lactation (EBF+) and followed up at 12 months postpartum (Fig 1). All births took place in the same maternity ward.

The project was approved by the Research Ethics Committee of the Faculty of Medicine of Botucatu (FMB-UNESP), under opinion 3,662,275/2019. Written informed consent was obtained from all women, who voluntarily participated in this study.

The inclusion criteria for both EBF and EBF+ were women aged between 18 and 38 years, who were in the postpartum period of their first birth (EBF: up to the 14th day, so that it was possible to carry out the exams scheduled on the 15th day, who were exclusively breastfeeding, and who intended to continue EBF until the infant’s sixth month of life, and EBF + ; who had exclusively breastfed their children for six months and, in addition, donated excess breast milk during this period, whose pregnancy resulted in a single newborn, full-term, with an appropriate weight for gestational age, who had no previous bone problems, and/or diseases that could compromise bone metabolism, and were not using medications that could promote the same effect. All the mothers continued breastfeeding their babies in a complementary way during the following 6 months.

Women were not included if they had a BMI above 30 kg/m^2^, a history of previous miscarriage, performed intense physical activities (at an athletic level or linked to the daily practice of systematic physical exercises), had used supplementation with calcium and/or vitamin D during pregnancy and the postpartum period, and those with chronic diseases, including diseases that presented alterations only in the hormonal profile.

Pregnant women who received prenatal care and were only attended by the Brazilian public health system (SUS), and mothers who were breastfeeding, were invited. There was no indication of calcium or vitamin D supplementation in either group, since this recommendation is not included in the protocol of the country’s Unified Health System. Pregnant and lactating women who receive supplemental or private health care receive guidance on supplementation as recommended by the Brazilian Federation of Gynecology and Obstetrics Associations and the Brazilian Society of Pediatrics [11,12] We considered these differences in monitoring, since the literature consulted often does not discriminate regarding vitamin or Calcium supplementation. The researchers recruited lactating women from the public health system who according to the recommended guidelines, use only folic acid and Ferrous Sulfate supplementation [13].

Women who did not provide consent and/or sign the informed consent form were excluded, as well as those who stopped exclusive breastfeeding before completing six months postpartum and those who stopped complementary breastfeeding belonging to the EBF+ cohort, those who did not attend on the days scheduled for biochemical, hormonal, and densitometric tests.

The sample calculation to establish the number of participants was carried out considering the study of Costa *et al*. [14], for a standard deviation of the distal radius of 0.0057 g/cm^2^, alpha of 5%, power of 90%, and difference between measurements of 0.010 (first measurement of 0.473g/cm^2^ and last measurement of 0.463 g/cm^2^), obtaining a sample n = 36 per research group. To compensate for possible losses or exclusion of participants, an additional 10% of postpartum women were included in each group.

To assess nutritional status, weight (kg) and height (m) were measured to calculate the Body Mass Index (BMI) (kg/m²). Pre-pregnancy weight was obtained by consulting the pregnant woman’s medical card and confirmed in one of the questions present in the general information questionnaire.

All the breastfeeding mothers (EBF and EBF+) responded to a questionnaire with general information, such as socioeconomic data (profession, family income, and level of education), medications in use, and frequency of consumption of foods rich in calcium and vitamin D.

Blood samples were collected, processed, and analyzed at the Carlos Chagas Laboratory, and Bone Densitometry exams were carried out at Dimen Diagnostico Médico Nuclear LTDA.

BMD (g/cm²) and Bone Mineral Content (BMC) (g) of the lumbar spine (L1-L4), total proximal femur (including femoral neck and trochanteric and intertrochanteric regions), and total body were performed by DXA on a GE-Lunar DPX NT device, with blinding of the technician and radiologist so that they would not know which group the breastfeeding mothers belonged to. Body fat mass, percentage of fat tissue, and amount of lean mass were also evaluated by DXA. The coefficient of variation (CV) was 0.86% for the lumbar spine, 1.39% for the femoral neck, and 1.00% for the total body.

Blood samples were obtained in the morning, between 7 and 9 am, after fasting for 8–10 hours, and collected by venipuncture, carried out by trained technicians from the contracted laboratory, and centrifuged for 15 min at 1500*g* for the separation of serum. The serum samples were stored at −70°C until the time of analysis of the bone turnover markers (OC, BAP, and S-CTX).

Osteocalcin (ng/mL), an indicator of bone formation, was quantitatively measured in serum using a Cobas model E 801 analytical device from Roche, performed using the sandwich technique, with the Elecsys N-MID Osteocalcin Kit from Cobas®. For the inter-trial values, with means of 6.7, 26.2, and 53.9 (ng/ml), respectively, the coefficients of variability were 5.1, 2.7, and 4.2%, and for the intra-assay, the coefficients were 1.3, 1.8, and 2.2%.

Bone alkaline phosphatase (µg/L) was measured using a LIAISON ® XL device, manufactured by DiaSorin through chemiluminescence, carried out by immunoassay, using the LIAISON® BAP OSTASE® kit. The coefficient of variation accuracies observed with 15.87, 21.51, and 31.14 µL were 4.7, 4.9, and 5.8%, respectively.

Carboxy-terminal telopeptide (S-CTX) (ng/mL) was measured in plasma (EDTA) with a Cobas model E 801 analytical device from Roche, using electrochemiluminescence technology, performed using the sandwich technique, with Elecsys β-CrossLaps Kit/Cobas® serum, adopting a coefficient of variation of 1.1%. Reference values (premenopausal women) were 0.025 to 0.573 ng/mL.

Calcium analysis (mg/dL) was carried out in serum, using a Lab max/Cobas® device, with a colorimetric method. Calcium reacts with o-cresolphthalein complexone (cfx) at pH 11, producing a dark red addition complex that is measured on a photocolorimeter at 570 nm. Duplicates of the same samples were processed on the same day, with levels of 7.10 and 15 mg/dL, and coefficients of variation of 1.7, 1.1, and 0.68%, respectively.

The 25-hydroxyvitamin D [25(OH)D] was measured in serum (ng/mL) with a Siemens Atellica IM Analyzer device, using chemiluminescence. The competitive immunoassay technique was used, employing the Atellica™ IM VitD kit from Siemens. This technique shows minimal cross-reactivity (<6%) of 3-epi, tested at 100 ng/mL, with a coefficient of variation of <10%. The recent suggestions of the Endocrine Society were used as a reference for analyzing the results [15,16].

Phosphorus (mg/dL) was measured in serum using the Lab max/Cobas® device. Inorganic phosphorus reacts in an acidic medium with molybdate to give a phosphomolybdic complex that is measured spectrophotometrically at 340 nm. Replicates of the same samples were processed on the same day, with levels of 3.6 and 7.5 mg/dL, and coefficients of variation of 2.64 and 2.18%, respectively.

The 17 β estradiol (pg/mL) was determined in serum using an enzyme-linked immunosorbent assay (ELISA) kit on a Vidas device. The competitive immunoassay technique was used, with the Atellica™ IM and E2 kit from Siemens. Samples were analyzed on an Atellica IM analyzer in duplicate, in 2 runs per day, for 20 days. The assay has a laboratory precision of ≤5.00 standard deviations for samples ≤25.00 pg/mL (91.75 pmol/L), and a coefficient of variation ≤7% for samples of 100.00–899.00 pg/mL (367.00–3299.33 pmol/L).

### Statistical analysis

The data were stored in an Excel spreadsheet, and analyzed using the SAS for *Windows* program, v.9.2., version 2013. The normality of the variables analyzed was verified using the Shapiro Wilk test. For statistical analysis, the paired Student t-test was used in comparisons of age, anthropometric variables (weight, height, BMI), hormonal variables, densitometric measurements, and bone turnover markers between exclusive breastfeeding mothers at 15 days and six months and the cohort EBF+ between six months and 12 months. To analyze the mean percentage differences in BMD at different sites between the 15-day and 6-month EBF timepoints, and EBF+ at six- and 12-months post-partum timepoints, the paired Student t-test was used. In all tests, the significance level was set at 5% or the corresponding p-value.

## Results

**Fig 2:**
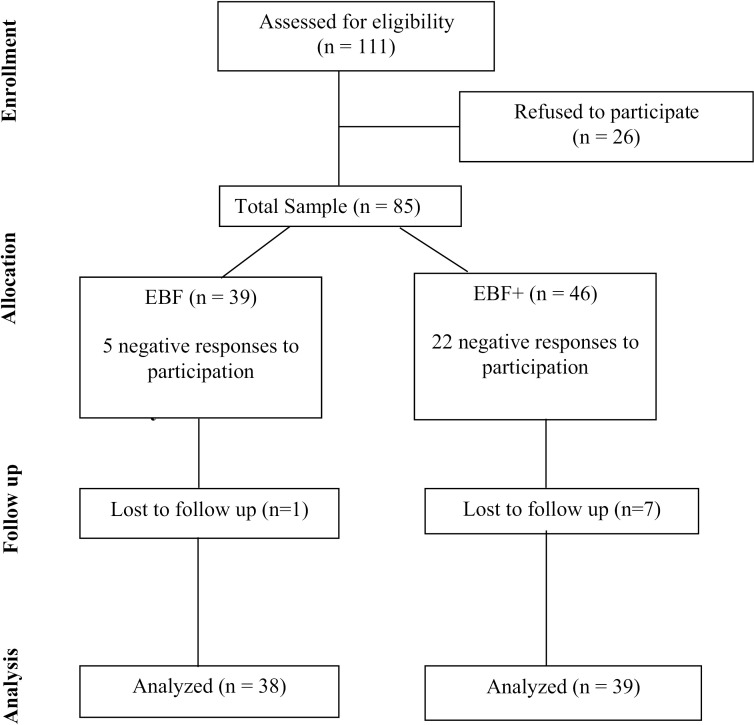
Flowchart of the exclusive breastfeeding mothers (EBF) and the exclusive breastfeeding mother-donors (EBF+) who participated in the study.

Initially, 111 women who met the established inclusion criteria were invited to participate in the study. Of the 43 women who met the criteria for the EBF group, 39 agreed to participate and 38 were analyzed. For the EBF+ group, of the 68 women invited, 46 agreed to participate and 39 were analyzed (Fig 2).

The 38 participating EBF had a mean age of 26.5 years ± SD 5.1 years, with minimum and maximum limits for inclusion of 18 and 37 years (Table 1). Considering the general characteristics of the mothers, 58.8% of births were by cesarean section, 64.9% had a secondary education level, 74.3% had professional occupations, 67.6% were married, and 54.1% had an average income of 1 to 3 minimum wages (Table 1). The results for the group of EBF + can also be seen in Table 1, highlighting that they differ statistically from the EBF in terms of the level of education and marital status.

**Table 1:** General and sociodemographic characteristics of the EBF and EBF+ groups.

Variables	EBF	EBF+	p-value
**Age** (years) Mean (SD)	26.5 ± 5.1	28.2 ± 5.4	0.211
**Pre-pregnancy**			
Weight (kg)	64.85 ± 11.97		
BMI (kg/m^2^)	26.13 ± 6.05		
**Type of Birth** (%)			
Vaginal	41.2	25.0	0.163
Cesarean	58.8	75.0	
**Schooling** (%)			
Fundamental	2.7	10.3	0.300
High school	64.9	33.3	0.010
Higher	32.4	56.4	0.060
**Work** (%)			
Yes	74.3	74.4	0.900
No	25.7	25.6	
**Marital status** (%)			
Married/Stable Union	67.6	97.4	0.020
Single	32.4	2.5	
**Ethnicity** (%)			
White	73.0	69.2	1.000
Mixed-race	27.0	30.8	0.737
**Income** (%)			
No salary specified	5.4	5.1	1.000
1 to 3*	54.1	51.5	0.510
3 to 6*	37.8	30.7	0.683
> 6*	2.7	12.7	0.226

* Minimum wage R$1100.00, ref. year 2021.

The anthropometric and nutritional characterizations of the EBF and EBF+ are presented in Table 2, which shows no significant variations in weight and BMI between the timepoints, for EBF or for EBF + . However, there were decreases in lean mass, fat mass, and fat percentage after six months for the EBF, and significant differences were identified between 6 and 12 months for EBF + .

**Table 2:** Anthropometric, nutritional, and variable assessments of lean mass and fat mass obtained by DXA of exclusive breastfeeding mothers (EBF), at pre-pregnancy, baseline (15 days), and six months and for exclusive breastfeeding-donors (EBF+) at 6 and 12 months.

Nutritional status										
Variables	EBF 15 days (n = 38)		EBF 6 months (n = 38)			EBF + 6 months (n = 39)		EBF + 12 months (n = 39)		
	mean ± SD	median	mean ± SD	median	p-value*	mean ± SD	median	mean ± SD	median	p-value*
Weight (kg)	68.08 ± 11.99	65.50	64.64 ± 11.72	60.50	0.338	65.9 ± 12.8	61.00	65.2 ± 12.8	63.00	0.421
Height (m)	1.61 ± 0.05					1.62 ± 0.5				
BMI (kg/m^2^)	26.20 ± 4.09	26.74	25.01 ± 3.86	24.35	0.210	25.10 ± 4.5	25.10	24.7 ± 4.7	24.50	0.208
Lean mass (kg)	36.36 ± 4.24	34.77	35.34 ± 3.94	34.50	0.017	35.06 ± 4.09	34.40	35.55 ± 3.54	34.76	0.015
Fat mass (kg)	27.80 ± 8.75	26.64	25.07 ± 8.95	24.03	<0.001	27.22 ± 9.52	23.91	25.53 ± 9.87	25.12	0.026
% fat	42.51 ± 6.99	42.50	40.35 ± 8.07	40.10	<.0001	42.56 ± 6.81	42.60	40.40 ± 9.16	42.00	0.012

Note: *Paired Student t-test, SD – Standard Deviation. BMI – Body Mass Index. g – Grams.

Table 3 presents the means, standard deviations, and medians of bone mineral density and content in the lumbar spine, total proximal femur, and total body, as well as biochemical, hormonal, and bone marker analyses (Ca, P, PTH, 25(OH)D, E2, BAP, OC, S-CTX). When comparing the variables obtained from the same mothers in the EBF group 15 days postpartum against their results after six months of EBF, a significant decline in BMD of the lumbar spine was observed at six months postpartum (1.075 g/cm2 ± 0.112) in relation to 15 days postpartum (1.107 g/cm^2 ^± 0.109), with *p* < 0.001.

**Table 3:** Comparisons between densitometric, biochemical, and hormonal variables and bone remodeling markers of exclusive breastfeeding mothers (EBF) 15 days postpartum and the same EBF mothers after six months.

	EBF 15 days postpartum (n = 38)		EBF 6 months postpartum (n = 38)		
	Mean ± SD	Median	Mean ± SD	Median	p*
Lumbar spine BMD (L1-L4) (g/cm2)	1.107 ± 0.109	1.115	1.075 ± 0.112	1.074	**<0.001**
T-score	−0.621 ± 0.924	−0.500	−0.835 ± 1.024	−0.800	**0.005**
Lumbar spine BMC L1-L4 (g)	55.31 ± 7.52	55.60	52.96 ± 7.89	53.57	**<0.001**
Total Body BMD (g/cm2)	1.135 ± 0.086	1.126	1.119 ± 0.085	1.104	**<0.001**
T-score	0.231 ± 0.995	0.100	0.181 ± 1.007	0.200	**<0.001**
Total Body BMC (g)	2465.1 ± 351.20	2418.00	2434.80 ± 366.60	2406.00	**0.005**
Femur BMD (g/cm2)	0.961 ± 0.105	0.974	0.929 ± 0.097	0.948	**<0.001**
T-score	−0.368 ± 0.833	−0.300	−0.522 ± 0.833	−0.200	**<0.001**
Femur BMC (g)	27.26 ± 4.48	27.17	26.37 ± 4.17	27.13	**0.011**
Ca (mg/dL)	9.09 ± 0.48	8.90	9.18 ± 0.55	9.10	0.407
P (mg/dL)	4.31 ± 0.70	4.20	4.12 ± 0.74	4.00	0.209
PTH (pg/mL)	24.64 ± 12.59	20.75	37.01 ± 15.92	34.65	**<0.001**
25(OH)D (ng/mL)	30.01 ± 11.68	28.80	27.36 ± 8.96	27.90	0.217
Estradiol (pg/mL)	51.33 ± 55.18	23.87	41.86 ± 46.91	22.50	0.503
BAP (mcg/L)	20.27 ± 11.76	17.70	21.41 ± 10.62	19.20	0.600
OC (ng/mL)	32.18 ± 11.59	29.50	36.86 ± 10.44	37.00	**0.017**
S-CTX (ng/mL)	1.01 ± 0.22	1.00	0.98 ± 0.48	1.00	0.540

Standard Deviation. g – Grams. BMD – Bone Mineral Density. BMC – Bone Mineral Content. PTH – Parathyroid Hormone BAP – Bone Alkaline Phosphatase. OC – Osteocalcin. S-CTX – Carboxy-terminal telopeptide

The results regarding total body BMD also showed decreases over the six months of EBF; at 15 days postpartum the mean was 1.135 g/cm^2^ ± 0.086, and at six months the mean was 1.119 g/cm^2^ ± 0.085 (**p* *< 0.001). This reduction was also observed in the total proximal femur, in which the first densitometry value indicated a mean of 0.961 g/cm^2^ ± 0.105 and at six months, a mean of 0.929 g/cm^2 ^± 0.097, p < 0.001.

Regarding bone turnover markers, a mean concentration of 32.18 ng/mL (SD ± 11.59) was observed for OC at baseline and 36.86 ng/mL (SD ± 10.44) at six months, with a significant difference, *p* = 0.017. Considering PTH, the results indicated an increase in the mean from 24.64 pg/mL to 37.01 pg/mL in the sixth month postpartum, with *p < *0.001. For the other biochemical and hormonal variables and bone turnover markers, no statistical differences were observed when comparing the timepoints (Table 3).

The mean percentage of BMD involvement was −3.4 ± 3.7% (p < 0.001) in the lumbar spine, −2.5 ± 3.4% (p < 0.001) in total proximal femur, and −1.7 ± 1.9% (p = 0.001) in total body, between EBF at 15 days postpartum and EBF at 6 months postpartum.

When analyzing Table 4, in the comparisons of the EBF+ cohort six months versus EBF + one year postpartum, it can be observed that for lumbar spine BMD (at six months the mean was 1.083 g/cm^2^** **± 0.121 and, at 12 months 1.138 g/cm^2^** **± 0.111 (p < 0.001), the mean for total femur BMD was 0.925 ± 0.129g/cm^2^ at six months and 0.945 ± 0.129 g/cm^2^ at 12 months (p = 0.001). No significant differences were detected for total body BMD. For the other variables analyzed (Ca, P, PTH, 25(OH)D, E2, BAP, OC, S-CTX), the significant differences are shown in Table 4.

**Table 4:** Comparison of densitometric, biochemical, and hormonal variables, and bone turnover markers of EBF + 6 months versus EBF + one year postpartum.

Variables	EBF + 6 months postpartum (n = 39)		EBF + 12 months postpartum (n = 38)		
	Mean ± SD	Median	Mean ± SD	Median	p*
BMD (g/cm^2^) L1-L4	1.083** **± 0.121	1.077	1.138** **± 0.111	1.156	**<0.001**
T-score	−0.726 ± 1.084	−0.800	−0.289 ± 0.917	−0.200	**<0.001**
BMC L1-L4 (g)	55.13 ± 9.68	53.91	59.08 ± 9.88	59.15	**<0.001**
Total body BMD (g/cm^2^)	1.135 ± 0.090	1.126	1.137 ± 0.084	1.126	0.348
T-score	0.265 ± 1.122	0.400	0.183 ± 1.04	0.000	0.773
Total body BMC (g)	2469.21 ± 363.35	2401.00	2480.02 ± 371.93	2392.50	0.376
Femur BMD (g/cm^2^)	0.925 ± 0.129	0.946	0.945± 0.129	0.960	**0.001**
T-score	−0.582 ± 1.036	−0.500	−0.5 ± 1.066	−0.450	**0.022**
Femur BMC (g)	26.61 ± 4.36	26.23	27.15± 4.53	27.31	**0.034**
Ca (ng/dL)	8.84^** **^± 0.84	9.00	9.19^** **^± 0.58	9.20	**0.037**
P (ng/dL)	6.39 ± 4.72	5.00	3.88 ± 0.57	4.00	**0.001**
PTH (pg/mL)	34.44 ± 18.29	32.2	45.18± 21.11 ^**c**^	40.50	**0.001**
25(OH)D(ng/mL)	23.75 ± 6.33	24.9	24.99 ± 10.81	24.10	0.515
Estradiol (pg/mL)	56.53 ± 52.97	36.5	104.12 ± 99.98	63.70	0.055
BAP (mcg/L)	20.78 ± 7.77	19.60	16.62± 7.05	15.20	**0.001**
OC (ng/mL)	33.89 ± 10.52	33.00	30.70 ± 11.85	29.00	0.090
S-CTX (ng/mL)	1.12± 0.40	1.14	0.631± 0.33	0.59	**<0.001**

***Note*:** * *Paired Student* t-test*.* SD – Standard Deviation. g – Grams. BMD – Bone Mineral Density. BMC – Bone Mineral Content. PTH – Parathyroid Hormone BAP – Bone Alkaline Phosphatase. OC – Osteocalcin. S-CTX – Carboxy-terminal telopeptide.

The mean percentage BMD increases were 5.0 ± 3.9% for lumbar spine and 1.6 ± 3.4% for total proximal femur. No significant differences were observed in the percentage variation in total body BMD, with a variation of 0.01 ± 2.1%.

## Discussion

As a result of exclusive breastfeeding, a decrease was observed in the BMD of lactating mothers in all sites evaluated, lumbar spine (L1-L4), total proximal femur, and total body. The decline in bone mass, obtained through DXA, was significant in the first six months of EBF, compared to the results evidenced in the postpartum period, which revealed a physiological mobilization of the bone mass targeted to the demands of calcium via breastmilk, necessary for the development and growth of newborns and infants. Regarding the cohort study carried out among exclusive breastfeeding-donors (EBF+) between six and 12 months, when performing complementary breastfeeding, significant increases were observed in the bone mineral density of the lumbar spine (L1-L4) and total proximal femur, but this was not identified for the total body BMD, and was accompanied by a significant reduction in the bone resorption marker S-CTX, during the six months of complementary breastfeeding.

The studies published to date, regarding the impact of breastfeeding on BMD [4,10,17–25], do not present similar designs to the current study, where only primiparous women were included, and who were breastfeeding mothers on EBF during the first six months postpartum, indicating that these breastfeeding mothers probably had an average production of milk/day of 800 mL or more [2].

Both pregnancy and lactation can be considered risk situations that impact the reproductive life of women in terms of identifying physiological mobilization of bone mass, however, the recovery of these alterations has not yet been definitively elucidated [10,21,26–34], indicating the need for further studies.

In order to investigate the relations of the mobilization evidenced during pregnancy and lactation, some authors carried out retrospective longitudinal studies, evaluating the results from DXA in women in the pre-menopausal and menopausal period, seeking to identify whether the decline in bone mass was associated with the history of pregnancies and period of breastfeeding carried out by these women throughout their reproductive life [4,17,23]. However, these analyses only reflected the timepoints analyzed and may or may not be associated with the pregnancy and lactation that occurred, as other situations experienced could result in decreases in bone mass, such as a history of medication use [35], physical activity [36], access to certain types of food [35], genetic predisposition [36], and the menopausal condition itself [36]. These studies concluded that the longer the duration of breastfeeding, the lower the BMD of the lumbar spine [4,20], with, probably, a greater impact on multiparous women [23], especially when they have not yet recovered their bone density and become pregnant sequentially, at short intervals [37].

The reductions observed in BMD and BMC, both in the lumbar spine, total proximal femur, and total body, although transient, were the result of effective adaptive responses, but could result in an increased risk to the bone health of lactating mothers, whether currently or in later periods of the life cycle, which has been contested in the literature. Furthermore, the consequences for bone mass if successive pregnancies occurred, followed by prolonged periods of lactation with reduced intervals between them, remains to be elucidated what.

Prentice demonstrated that mothers from Gambia, with very restrictive diets regarding calcium intake (<400mg/day) and diets lacking other macro and micronutrients, who started breastfeeding around 14 years of age and who had multiple pregnancies (±7 pregnancies per woman) presented a lower BMC than breastfeeding mothers of adequate socioeconomic status from Cambridge. However, when they reached ages over 40, the BMC results overlapped with those of the Cambridge breastfeeding mothers [38]. Based on other studies, it was reported that although profound changes occurred, they would be partially reversible over time, with long lactation or with weaning, resulting from hormonal mechanisms that are not fully understood [39].

Regarding the sociodemographic characteristics of the EBF and EBF+ participants, they were in a more favorable situation when compared to the average demonstrated in recently promulgated results regarding the Brazilian population [39]. The participants generally had secondary and higher education, were in the job market, and received an average of three minimum wages (Table 1). These results are related to their being residents of the Southeast region of the country, living in the state of São Paulo, which has the second highest Human Development Index (HDI) in the country, indicating that in addition to direct advantages they could benefit from other advantages considered as indirect, such as basic sanitation, social facilities, public services, environmental quality, and health and educational establishments, which are made available to populations residing in places with a higher HDI [40]. In addition, prior to inclusion, the knowledge of the mothers about the importance of EBF and about not introducing food and liquids early was assessed, which made the sample suitable for the development of the study.

The breastfeeding mothers participating in the current research were mostly eutrophic in the pre-gestational period and, according to the nutritional assessment, they remained so until the end of the follow-up. Several mechanisms have been proposed to explain the complex relationship between adipose tissue and bone, since adipose tissue secretes inflammatory cytokines, such as interleukin 6 (IL-6), IL-1, and tumor necrosis factor alpha (TNF-α), which promote bone resorption by stimulating the differentiation of osteoclasts [41,42].

The results from the present study showed a decline in bone mass resulting from six months of EBF. Confirming these results, a systematic review followed by meta-analysis indicated that there is a transient reduction in bone mineral density in the lumbar region of women who breastfeed their children exclusively for 4 to 6 months [10]. However, few have shown a reduction in BMD in other sites, total body, and total proximal femur. In the systematic review by Grizzo *et al.* [43], with the inclusion of five studies, femoral neck BMD assessments did not show a significant association between final and initial values. Among the few studies demonstrating this involvement, Kalkwarf and researchers [18] detected a reduction in BMD in the total body and Moller *et al.* [34] observed a 1% decline in the total body BMD, while in the lumbar spine the reduction was 5%. There is a consensus in the relevant literature about the greater impact of lactation on BMD of the lumbar spine, possibly because the other locations analyzed are made up of a greater proportion of cortical bone, considered metabolically less active when compared to trabecular bone, which are predominant in vertebrae [10].

At six months postpartum, an increase in maternal PTH levels was observed when compared to those documented at baseline. The increase in PTH promotes differentiation and activation of osteoclasts to increase the supply of Ca, resulting from activated osteoclastic resorption, which promotes a reduction in bone mass [10,25]. It is known that PTH acts in conjunction with hypoestrogenism, evidenced during the period of exclusive breastfeeding, which follows the increase in prolactin, promoting bone resorption and the activity of osteoclasts [9].

Miyamoto *et al.* [44] demonstrated that E2 levels were lower in postpartum women, when compared to premenopausal and menopausal women, stating that the postpartum period would be comparable to a “transient menopause”. Assessments of 17 β estradiol levels obtained 15 days postpartum and at six months resulted in values considered quite low, similar to those seen in the follicular phase of a mature woman’s menstrual cycle. At the 12 months of EBF + the concentrations had doubled comparing to EBF+ at six months postpartum (Table 4).

With respect to 25(OH)D, researchers have demonstrated that pregnant and lactating women may have concentrations whose limits are at least between 20–40 ng/mL, which would be considered appropriate, since concentrations below 20ng/mL are identified as suboptimal for bone health and may be associated with increased PTH levels [15,16]. Elevated PTH promotes bone resorption, leading to a decrease in BMD.

Cooke-Hubley *et al.* [24], evaluated 31 women at four to six months of breastfeeding and again at 12 months, when the mothers performed complementary breastfeeding and, even though they continued breastfeeding their children, their lumbar spine BMD increased by 4–5%, without increases in femoral neck and total body BMD, results close to those evidenced in the EBF+ cohort between 6 and 12 months in the present study, which in addition to being identified in the lumbar spine was also identified in the total femur BMD.

Considering the biochemical analyses, it was evident that all bone turnover markers analyzed (BAP, OC, and S-CTX) presented lower concentrations at 12 months, when considering the analysis of the EBF+ group. The reduction in bone turnover markers was possibly associated with a reduction in maternal bone resorption, since during this period of complementary breastfeeding the infant is no longer fed exclusively, with a consequent decrease in milk production and less Ca transferred via breast milk, added to the finding of an increase in estradiol concentrations in EBF+ at 12 months compared to the concentrations identified at six months.

One of the limitations of this study is that bone densitometry was not obtained from the lactating mothers prior to pregnancy, which would make it possible to determine the loss of bone mass that occurred not only during this period, but also to identify, at 12 months postpartum, the real reduction in bone mass compared to the same women when non-pregnant, and how much of this percentage is recovered at the end of a reproductive cycle of pregnancy and lactation. Another event that interfered with the conduct of this study was the outbreak of the COVID-19 pandemic, which resulted in high morbidity and mortality among pregnant and postpartum women across the country, preventing them from undergoing routine exams, and enabling only those that are strictly necessary.

The current study presents a great contribution, as no studies on this topic were detected with the specified inclusion criteria adopted, differentiating it from other published research. Thus, further studies are needed that include monitoring for longer periods, in different conditions, such as overweight/obese lactating mothers, with Ca and vitamin D supplementation, to determine additional factors that may lead to or result in a reduction in impacts, which although transitory, can act on the bone mass of lactating mothers.

## Conclusions

It is concluded that after six months of EBF, there is a significant physiologic adaptation in bone mineral density in the lumbar spine, total body, and total proximal femur. At 12 months, analyzing the EBF+ cohort, even if breastfeeding continues in a complementary form, densitometric results tend to increase to higher and significant values compared to those observed in the same EBF+ at 6 months, whether in the lumbar spine or in the total femur, with the exception of total body BMD. Exclusive breastfeeding is an integral part of the recommendations proposed by the WHO and public health policies and is part of the national humanization policy. Therefore, monitoring postpartum women and not informing them about the numerous benefits resulting from EBF seems inconceivable.

## Supporting information

S1 Open data. (XLSX)
